# Nestin Protein Is Phosphorylated in Adult Neural Stem/Progenitor Cells and Not Endothelial Progenitor Cells

**DOI:** 10.1155/2012/430138

**Published:** 2012-09-13

**Authors:** Jun Namiki, Sayuri Suzuki, Takeshi Masuda, Yasushi Ishihama, Hideyuki Okano

**Affiliations:** ^1^Department of Emergency and Critical Care Medicine, Keio University School of Medicine, Shinanomachi 35, Shinjuku, Tokyo 160-8582, Japan; ^2^Institute for Advanced Biosciences, Keio University, 403-1 Daihoji, Tsuruoka, Yamagata 997-0017, Japan; ^3^Graduate School of Pharmaceutical Sciences, Kyoto University, Kyoto 606-8501, Japan; ^4^Department of Physiology, Keio University School of Medicine, Shinanomachi 35, Shinjuku, Tokyo 160-8582, Japan

## Abstract

An intermediate filament protein, Nestin, is known as a neural stem/progenitor cell marker. It was shown to be required for the survival and self-renewal of neural stem cells according to the phenotypes of Nestin knockout mice. Nestin expression has also been reported in vascular endothelial cells, and we recently reported Nestin expression in proliferating endothelial progenitor cells, but not in mature endothelial cells. Using quantitative phosphoproteome analysis, we studied differences in phosphorylation levels between CNS Nestin in adult neural stem cells and vascular Nestin in adult bone-marrow-derived endothelial progenitor cells. We detected 495 phosphopeptides in the cell lysates of adult CNS stem/progenitor cells and identified 11 significant phosphorylated amino acid residues in the Nestin protein. In contrast, endothelial progenitor cells showed no significant phosphorylation of Nestin. We also measured neoplastic endothelial cells of the mouse brain and identified 13 phosphorylated amino acid residues in the Nestin protein. Among the 11 phosphorylated amino acids of adult CNS Nestin, five (S565, S570, S819, S883, and S886) were CNS Nestin-specific phosphorylation sites. Detection of the CNS-specific phosphorylation sites in Nestin, for example, by a phospho-specific Nestin antibody, may allow the expression of CNS Nestin to be distinguished from vascular Nestin.

## 1. Introduction

Nestin is a class VI intermediate filament protein expressed in undifferentiated central nervous system (CNS) cells during development. The protein is known as a neural stem/progenitor cell marker and required for the survival and self-renewal of neural stem cells (NSCs) [1]. Nestin expression is downregulated when CNS stem/progenitor cells differentiate into neurons or glial cells [2, 3], and the expression is kept in adult CNS stem/progenitor cells that reside in the forebrain neurogenic regions [4, 5]. Nestin expression has also been reported in vascular endothelial cells (ECs) from a variety of adult human non-CNS tissues [6, 7]. We recently reported Nestin expression in proliferating endothelial progenitor cells (EPCs), but not in mature ECs [8]. We utilized E*/nestin* : EGFP transgenic mice using its second intronic enhancer element to study neural-specific *nestin* gene expression [9, 10] and demonstrated that vascular *nestin* expression is not activated by the CNS-specific enhancer of the *nestin* gene [8]. This finding indicated that the Nestin expressed in EPCs is cytochemically similar to the protein expressed in CNS stem/progenitor cells, but the regulatory mechanism of gene expression is different.

The reversible phosphorylation of proteins results in a conformational change that alters their function. Many proteins, including cellular receptors, enzymes, and intracellular signaling molecules, are activated/deactivated by phosphorylation/dephosphorylation. Thus, reversible phosphorylation plays a significant role in the regulation of cellular processes. Nestin protein in the cytoplasm of CNS stem/progenitor cells is thought to play a role in distributing Vimentin from copolymerized intermediate filaments to daughter cells during cell division [11]. Elevated phosphorylation of Nestin has been observed to accompany the mitotic reorganization of Nestin in an immortalized CNS precursor rat cell line [12]. Concerning the developing mouse brain, more than 500 phosphorylation sites on proteins, including Nestin, have been identified by phosphoproteomic analysis [13]. However, Nestin phosphorylation has not been investigated in adult NSCs. Using quantitative phosphoproteome analysis, we demonstrate in the present study that different phosphorylation levels are found among CNS Nestin in adult NSCs and vascular Nestin in adult bone-marrow-derived EPCs.

## 2. Materials and Methods

### 2.1. Animals

Adult (8 to 10 weeks old) wild-type C57BL/6J mice were purchased from SLC (Shizuoka, Japan). All animal-related procedures were approved by the Laboratory Animal Care and Use Committee of Keio University and conducted in accordance with the guidelines of the National Institutes of Health, USA.

### 2.2. Primary Neural Stem/Progenitor Cell Culture

Neurospheres were generated from adult mouse forebrain as described previously [8, 10, 14, 15]. Briefly, the striata from adult mice were dissected, incubated with trypsin solution for 15 min at 37°C, triturated, and then trypsin inhibitor solution added. Dissociated cells (5000 cells/mL) were seeded in neurosphere culture medium composed of DMEM-F12 (1 : 1), glucose (0.6%), glutamine (2 mM), sodium bicarbonate (13.4 mM), HEPES (5 mM), insulin (25 *μ*g/mL), transferrin (100 *μ*g/mL), progesterone (20 nM), sodium selenate (30 nM), and putrescine (60 nM) supplemented with recombinant human epidermal growth factor (EGF, 20 ng/mL) and recombinant human basic fibroblast growth factor (bFGF, 20 ng/mL). Cells were cultured for 7 days *in vitro* (DIV) and formed floating cell clusters of neural stem/progenitor cells (neurospheres). Primary neurospheres were collected and mechanically triturated. Dissociated cells were counted and stored at −20°C.

### 2.3. EPC Culture

EPCs were cultured from mononuclear cells (MNCs) under previously reported culture conditions [8]. The femurs and tibias of adult mice were crushed, suspended in *α*MEM (#11900, Gibco Invitrogen, Carlsbad, CA) supplemented with 10% fetal bovine serum (FBS) and 1% penicillin G (10,000 units/mL) streptomycin sulfate (10,000 *μ*g/mL) (PS), and filtered through a 70-*μ*m filter (Cell Strainer #352350, Falcon, Bedford, MA). MNCs were isolated from bone marrow cells by Ficoll density-gradient centrifugation (Ficoll-Paque Plus, 1.077 g/mL, GE Healthcare, Uppsala, Sweden). Cells (1 × 10^6^ cell/mL) were plated on fibronectin-coated 6-well plates (#140675, Nunc, Roskilde, Denmark) in endothelial basal medium supplemented with 5% FBS, vascular endothelial growth factor (VEGF), bFGF, recombinant analog of insulin-like growth factor-1 (R^3^-IGF-1), EGF, hydrocortisone, ascorbic acid, and gentamicin/amphotericin-B (EGM-2-MV Bullet Kit CC-3202, Lonza, Walkersville, MD). The medium was changed after 24 hours to remove nonadherent cells and renewed every week. At 21 DIV, cells were lifted by incubation with 0.25% trypsin and 1 mM EDTA and then stored at −20°C.

Although a unique EPC marker has not been identified, EPCs are characterized as cells with a high proliferative potential that display typical endothelial characteristics and differentiate into ECs *in vitro* [16]. EPCs obtained under the above culture conditions were positive for the proliferation marker Ki67, positive for EC lineage marker CD31 and vascular endothelium cadherin or the uptake of 1,1′dioctadecyl- 3,3,3′,3′-tetramethylindo-carbocyanine perchlorate Ac-LDL (DiI–Ac-LDL), negative for the mature EC marker von Willebrand factor (vWF), and capable of differentiating into mature ECs [8].

### 2.4. EC Line

To compare phosphorylation between CNS Nestin and vascular endothelial Nestin in proliferative cells further, we prepared neoplastic ECs. Cells from a mouse brain endothelioma cell line (bEnd.3 cells CRL-2299, ATCC, Manassas, VA) were characterized as proliferative endothelial cells expressing vascular Nestin similar to EPCs and positive for mature EC marker vWF [8].

The EC line was cultured according to the manufacturer's instructions. Briefly, cells were maintained in DMEM (#12699, Gibco Invitrogen) supplemented with 10% FBS and 1% PS. The medium was renewed every 3 to 4 days. Cells were harvested by incubation with 0.25% trypsin and 1 mM EDTA and stored at −20°C.

### 2.5. Quantitative Phosphoproteome Analysis

Cells were processed for phosphoproteome analysis based on mass spectrometry (MS) coupled with miniaturized on-line liquid chromatography (LC). Proteins were extracted from cells (100,000 cells from adult neurospheres; 1,000,000 cells from EPCs and the neoplastic ECs) using 12 mM sodium deoxycholate and 12 mM sodium lauroyl sarcosinate, and digested with Lys-C and trypsin [17]. Phosphopeptides were enriched by aliphatic hydroxy acid-modified metal oxide chromatography with titania [18] and analyzed by nanoLC-MS/MS using an LTQ-Orbitrap instrument (Thermo Fisher Scientific, Bremen, Germany). Peptides and proteins were identified using Mascot version 2.3 (Matrix Science, London) with the SwissProt database. Label-free quantitation was performed based on the peak areas of extracted ion chromatograms for identified phosphopeptides using Mass Navigator (Mitsui Knowledge Industry, Tokyo, Japan).

## 3. Results

### 3.1. Protein Phosphorylation of CNS Stem/Progenitor Cells, EPCs, and Neoplastic ECs

CNS stem/progenitor cells were obtained from striata of the lateral wall of the lateral ventricles in the adult mouse brain and grown as neurospheres *in vitro*. Phosphoproteome analysis detected 495 phosphopeptides in the cell lysates (Table 1). Approximately 90% of the peptides detected in neurosphere cells were phosphorylated. A similar percentage of phosphopeptides was measured in neoplastic ECs. However, approximately 60% of peptides in nonneoplastic proliferative endothelial cells, EPCs, were phosphorylated, indicating that intracellular and cell membrane proteins were activated overall in neurosphere cells compared to EPCs. Generally, phosphorylation occurs most commonly on serine, followed by threonine. More than 80% of phosphorylated sites were serine residues in our samples, and the ratio of phosphorylated amino acids was not different between neurosphere cells, EPCs, and neoplastic ECs (Table 1). We tabulated phosphorylated proteins and their phosphorylated amino acid residues in Figure 1 in Supplementary Material (available online at doi:10.1155/2012/430138).

### 3.2. Nestin Phosphorylation of CNS Stem/Progenitor Cells, EPCs, and Neoplastic ECs

**Figure 1:**
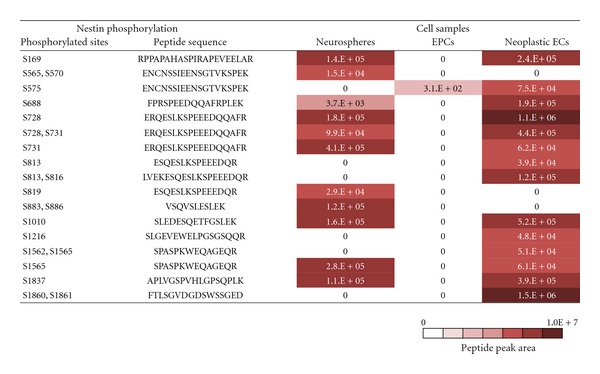
Quantitative phosphoproteome analysis of Nestin. A peak area of more than 1.E + 04 indicates significant phosphorylation detected by MS/MS. pSites, phosphorylated sites of amino acid residues.

Quantitative phosphoproteome analysis identified 10 phosphopeptides and 11 significant phosphorylated amino acid residues (a peak area >1.E + 04) in the Nestin protein from adult neurosphere cells (Figure 1). In contrast, EPCs derived from adult mouse bone marrow showed no significant phosphorylation of Nestin (*n* = 2). In neoplastic ECs, 13 significant phosphorylated amino acid residues were identified. Thus, the finding that Nestin is not phosphorylated in EPCs is not likely due to the tissue specificity of ECs. All phosphorylated amino acid residues found in neurosphere cells and neoplastic ECs were serine. Although phosphothreonine was reported in samples of Nestin from an immortalized rat cell line [12] and human HeLa cells (cervical carcinoma) [19, 20], we did not detect the phosphorylation of threonine residues in the Nestin proteins from our samples. Five of the phosphorylated amino acid residues (S565, S570, S819, S883, and S886) were detected in neurosphere cells only, eight (S575, S668, S813, S816, S1216, S1562, S1860, and S1861) were detected in neoplastic ECs only, and six (S169, S728, S731, S1010, S1565, and S1837) were detected in both neurosphere cells and neoplastic ECs (Figure 1).

In CNS stem/progenitor cells, Nestin protein preferentially forms heterodimers and heterotetramers with a variety of intermediate filament proteins, particularly type III Vimentin and type IV *α*-Internexin [21, 22]. Phosphoproteome analysis detected phosphorylation of Vimentin but not *α*-Internexin from the samples of adult neurospheres, EPCs, and neoplastic ECs (Figure 1 in Supplementary Material).

## 4. Discussion

Recent investigations of *nestin*-knockout mice have reported that Nestin deficiency results in embryonic lethality with the neuroepithelium of the developing neural tube exhibiting low numbers of NSCs and high levels of apoptosis [1]. The downregulation of* nestin* in the embryonic cerebral cortex using small interference RNAs against *nestin* mRNA results in G1 cell-cycle arrest and a severe reduction in the generation of neurons [23]. However, no data have been reported on the *in vivo* function of adult Nestin. Transient transfection of *nestin-*non-expressing cells with an expression vector carrying rat *nestin *cDNA has been shown to promote the disassembly of phosphorylated Vimentin intermediate filaments in the cytoplasm during mitosis [11]. Our phosphoproteome analysis detected phosphorylated Vimentin in the adult neurosphere sample. Thus, Nestin in adult NSCs is likely to mediate the distribution of Vimentin protein to daughter cells during self-renewal and neurogenesis.

In a rat neuronal progenitor cell line, the mitotic reorganization of Nestin was accompanied by the elevated phosphorylation of Nestin, and T316 was identified as a Nestin phosphorylation site [12]. Phosphorylated threonine was not detected in Nestin from adult CNS stem/progenitor cells, EPCs, or neoplastic ECs in the present study. However, we identified 11 significant phosphorylation sites at serine residues in Nestin protein from adult CNS stem/progenitor cells using quantitative phosphoproteome analysis. The difference in phosphorylated amino acids and the number of phosphorylated sites may be due to technological advances in phosphoproteome analysis and/or the difference in cell sources. Phosphorylated serine residues have been reported in Nestin protein from the brain of mouse embryos [13] and mouse skin melanoma [24] and have been assumed by similar data in human HeLa cells [19, 20]. Among the 11 phosphorylated serine residues we identified in Nestin protein, only two (S565 and S1010) were reported previously; the other nine (S169, S570, S728, S731, S819, S883, S886, S1565, and S1837) were newly identified in the present study.

Nestin protein is expressed not only in NSCs, but also in tissue stem/progenitor cells beyond the germ layers, including mesenchymal stem cells [25], vascular endothelium [6–8], muscle [26–28], testes [29], and teeth [30]. Nestin is also abundant in progenitor cells derived from embryonic stem cells that have the potential to develop into neuroectodermal, endodermal, and mesodermal lineages [31]. We recently reported that Nestin is expressed in proliferating ECs and may be useful as a marker of neovascularization [8]. Nestin expression has been reported in the angiogenic endothelium of cancers [32, 33]. Independent cell-type-specific elements of the *nestin* gene are identified in transgenic mice; the first intron directs reporter gene expression to the mesodermal somite, and the second intron contains enhancer that functions in NSCs [28]. Although the regulatory mechanisms underlying *nestin* gene expression in proliferative vascular cells are different from those in NSCs, the protein expression of vascular Nestin is cytochemically similar to CNS Nestin [8]. The phosphorylation of Nestin protein from adult neurospheres can allow it to be distinguished from Nestin in EPCs. Although Nestin phosphorylation was also observed in neoplastic ECs, our phosphoproteome analysis identified CNS-specific phosphorylation sites, suggesting that a phospho-specific Nestin antibody may distinguish between the expression of CNS and vascular Nestin proteins.

## 5. Conclusions

Quantitative phosphoproteome analysis identified phosphorylated serine residues in Nestin from adult mouse CNS stem/progenitor cells. Phosphorylation was not observed in Nestin from EPCs. Detection of the CNS-specific phosphorylation sites in Nestin, for example, by a phospho-specific Nestin antibody, may allow the expression of CNS Nestin to be distinguished from vascular Nestin.

## Supplementary Material

Figure 1 in Supplementary Material: A list of phosphorylated sites of proteins identified in CNS stem/progenitor cells, EPCs, and neoplastic ECs. "1" in the list means that the phosphopeptide has been identified.

## Figures and Tables

**Table 1:** Protein phosphorylation of adult CNS stem/progenitor cells, EPCs, and neoplastic ECs.

	pPeptides (pPeptides/total peptides)	pSites			Multi-pPeptides		
		(pSite/total pSites)			(single or multi-pSite peptides/total pPeptides)		
		Serine	Threonine	Tyrosine	1p	2p	>2p
Neurospheres	495 (91.2%)	443 (84.2%)	71 (13.5%)	12 (2.3%)	401 (81.0%)	74 (14.9%)	20 (4.0%)
EPCs	250 ± 5 (59.7%)	194 ± 8 (87.4%)	20 ± 3 (9.0%)	8 ± 0 (3.6%)	228 ± 6 (91.0%)	19 ± 0 (7.4%)	4 ± 0 (1.6%)
Neoplastic ECs	980 (97.8%)	896 (88.1%)	103 (10.1%)	18 (1.8%)	675 (68.9%)	256 (26.1%)	49 (5.0%)

pPeptides, phosphopeptides; pSites, phosphorylation sites; multi-pPeptides, multi-phosphorylated peptides; 1p, single-phosphorylated site; 2p, two phosphorylated sites. Data are mean ± standard deviation.
